# The Safety and Efficacy of Left Atrial BOX Ablation in Persistent Atrial Fibrillation: A Meta-Analysis

**DOI:** 10.31083/j.rcm2509316

**Published:** 2024-09-06

**Authors:** Yang Li, Yin Xi, Wenyu Zhang, Jie Hao

**Affiliations:** ^1^Department of Cardiology, The Second Hospital of Hebei Medical University, 050000 Shijiazhuang, Hebei, China

**Keywords:** additional left atrial BOX ablation, persistent atrial fibrillation, catheter ablation, meta-analysis

## Abstract

**Background:**

Circumferential pulmonary vein isolation (CPVI) has a high recurrence rate in managing persistent atrial fibrillation (AF). While some studies suggest that augmenting CPVI with additional left atrial BOX ablation can diminish this recurrence rate among patients with persistent AF, this approach remains controversial. This meta-analysis assesses the safety and efficacy of adjunctive left atrial BOX ablation in treating persistent atrial fibrillation.

**Methods:**

We conducted a comprehensive literature search across China National Knowledge Infrastructure (CNKI), PubMed, Web of Science, and Cochrane Library, focusing on randomized controlled trials. The primary outcome was the recurrence rate of any atrial arrhythmias (AAs) within one-year post-treatment, with the secondary outcome being the frequency of adverse events related to the surgery.

**Results:**

The combination of CPVI and left atrial BOX ablation did not lead to a significant reduction in the overall recurrence rate of atrial arrhythmias (risk ratios (RR) = 0.86, 95% confidence interval (CI) = 0.73–1.02, I^2^ = 35%). However, subgroup analyses revealed that this therapeutic approach significantly decreased the recurrence rates of all atrial arrhythmias (RR = 0.67, 95% CI = 0.49–0.92, I^2^ = 15%) and specifically atrial fibrillation (RR = 0.53, 95% CI = 0.37–0.77, I^2^ = 0%) in patients with a left atrial diameter ≤44 mm. Notably, there was no significant increase in the incidence of procedure-related adverse events (RR = 1.04, 95% CI = 0.56–1.94, I^2^ = 0%). However, the durations of both the ablation (mean difference (MD) = 19.77, 95% CI = 15.84–23.70, I^2^ = 0%) and the overall procedure (MD = 15.64, 95% CI = 6.99–24.29, I^2^ = 0%) were longer due to the additional ablation steps.

**Conclusions:**

In patients with smaller left atrial diameters, augmenting CPVI with left atrial BOX ablation significantly lowers the recurrence rates of atrial arrhythmias and atrial fibrillation without elevating surgical risk levels.

## 1. Introduction

Atrial fibrillation (AF), a significant contributor to increased all-cause mortality [1], is associated with a heightened risk of stroke, heart failure, myocardial infarction, and cognitive decline. The mean annual hospitalization rate for AF patients is notably higher than that for individuals without AF [2]. Beyond impacting quality of life and health, AF imposes considerable economic and physical burdens. Catheter ablation, recognized as an effective strategy for managing AF, is endorsed in clinical guidelines as the primary treatment for individuals with paroxysmal AF and is also recommended for patients with persistent AF who respond poorly to antiarrhythmic medications [3, 4].

Although the electrophysiological mechanisms underlying AF require further investigation, it is widely accepted that ectopic beats emanating from the pulmonary veins frequently trigger AF episodes. Consequently, circumferential pulmonary vein isolation (CPVI) has emerged as a fundamental technique in AF ablation [5].

While CPVI is effective for paroxysmal AF, the recurrence rate remains high for persistent AF. Some initial studies have suggested that adding linear ablation outside the pulmonary veins could reduce recurrence in persistent AF ablation; however, the STAR AF II study challenged the efficacy of this additional procedure [6]. Recent advancements, such as the smart touch catheter and ablation index, have prompted further research into the benefits of additional linear ablation. The left atrial posterior wall, closely linked with the pulmonary veins, is often the site of significant non-pulmonary vein triggers in persistent AF [7]. Left atrial BOX ablation, which can isolate posterior left atrial (LA) by linear ablation of LA roof and bottom after PVI, could preventing AF triggers and help maintaining sinus rhythm [3]. Despite published randomized controlled trials (RCTs) on left atrial BOX ablation, the advantages of this additional procedure in patients with persistent AF remain debated. This meta-analysis seeks to assess the effects of supplementary left atrial BOX ablation on the recurrence and safety of persistent AF, to determine the safety and efficacy of left atrial BOX ablation, and to provide evidence supporting the use of catheter ablation to reduce recurrence rates in patients with persistent AF.

## 2. Methods

### 2.1 Inclusion and Exclusion Criteria

This review strictly included only RCTs. The experimental group comprised patients undergoing CPVI combined with left atrial BOX ablation, while the control group received CPVI alone. Eligible participants were those with persistent atrial fibrillation, diagnosed according to internationally recognized guidelines, and undergoing radiofrequency ablation for the first time. Exclusions were made for studies with incomplete data or duplications. Outcome measures included the recurrence rate of all postoperative atrial arrhythmias (including atrial fibrillation, atrial tachycardia, and atrial flutter), the incidence of surgery-related adverse events, ablation duration, and operative time.

### 2.2 Search Strategy 

The literature search encompassed China National Knowledge Infrastructure (CNKI), PubMed, Web of Science, and Cochrane Library, focusing on RCTs using the following terms: (“atrial fibrillation” OR “AF”) AND (“posterior wall” OR “posterior left atrium” OR “box”) AND (“ablation” OR “isolation”). The search timeframe extended from the inception of each database until January 2023. Two independent researchers screened the articles based on predefined exclusion criteria. Eligible studies were then thoroughly reviewed to select the final set of included papers. All included studies were strictly RCTs, and their quality was assessed using the Cochrane risk of bias tool.

### 2.3 Extraction Data 

The extracted data included publication details (year, title, first author); participant demographics (sample size, gender distribution, mean age, left atrial diameter, duration of atrial fibrillation); study specifics (intervention details, ablation techniques, catheter types, rates of all atrial arrhythmias and atrial fibrillation recurrence, incidence of procedure-related adverse events, ablation and operative durations).

### 2.4 Statistical Analysis 

This meta-analysis used R version 4.1.2 (R Foundation for Statistical Computing, Vienna, Austria), displaying findings in forest plots. Heterogeneity among the studies was evaluated using I^2^ statistics. A random-effects model was applied when I^2^ exceeded 50%; otherwise, a fixed-effect model was utilized. The analysis was further supplemented by conducting sensitivity analyses. Dichotomous outcomes were reported as risk ratios (RRs) with 95% confidence intervals (CIs), while continuous data were summarized using weighted mean differences (WMDs). A *p*-value of <0.05 denoted statistical significance.

## 3. Results

### 3.1 Literature Search and Selection Process

**Fig. 1.**
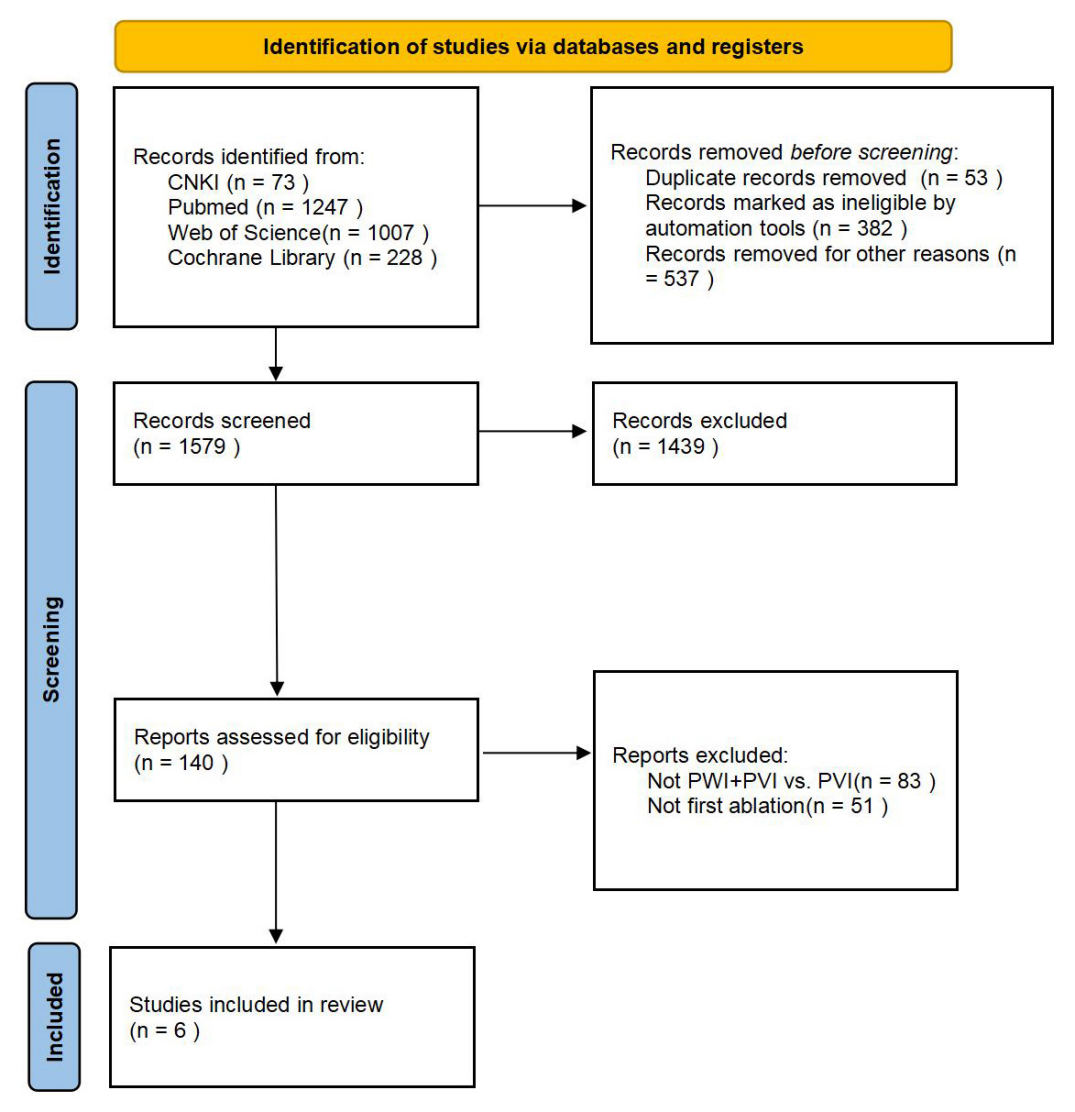
**The literature search process**. CNKI, China National Knowledge Infrastructure; PWI, posterior wall isolation; PVI, pulmonary vein isolation.

Initially, the search yielded 2554 records. After removing duplicates, screening titles and abstracts, and full-text reviews, six RCTs were ultimately included [8, 9, 10, 11, 12, 13] (Fig. 1).

### 3.2 Quality Assessment

**Fig. 2.**
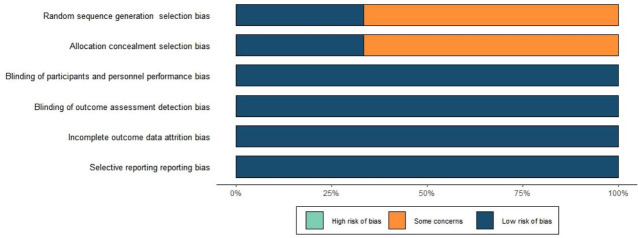
**Literature quality assessment results**.

We conducted quality assessment by taking the following aspects into account: (1) random sequence generation (selection bias), (2) allocation concealment (selection bias), (3) blinding of participants and personnel (performance bias), (4) blinding of outcome assessment (detection bias), (5) incomplete outcome data (attrition bias), (6) selective reporting (reporting bias), and (7) other bias (Fig. 2). The risk of publication bias was deemed to be low.

### 3.3 Baseline Characteristics

This study encompassed 967 patients diagnosed with persistent atrial fibrillation. Of these, 468 received CPVI combined with BOX ablation, while 499 underwent CPVI alone; 731 (75.6%) were male. Five studies employed radiofrequency (RF) ablation, whereas the study by Aryana *et al*. [12] combined cryoballoon and RF ablation. Notably, only the study by Kistler *et al*. [13] detailed the ablation index (AI) parameters during RF ablation and introduced a strategy of high-power, short-duration ablation. The six studies incorporated into this analysis conducted CPVI followed by additional linear ablation procedures, including the mitral isthmus line, tricuspid isthmus line, and anterior wall lines. The baseline characteristics of the study cohorts are delineated in Table 1 (Ref. [8, 9, 10, 11, 12, 13]), with specific details on the ablation procedures outlined in Table 2 (Ref. [8, 9, 10, 11, 12, 13]).

**Table 1.** **Patients’ baseline characteristics**.

Author	Group	Number	Male (%)	Age (year)	BMI (kg/m²)	LAD (mm)	Duration of AF (Mon)
Kim *et al*. 2015 [8]	Total	120	87 (72.5)	NA	NA	42.2 ± 5.8	NA
	CPVI + BOX	60	46 (76.7)	56.2 ± 11.9	24.4 ± 2.8	42.3 ± 6.4	NA
	CPVI	60	41 (68.3)	58.3 ± 9.6	24.4 ± 4.1	42.1 ± 5.1	NA
Lee *et al*. 2019 [9]	Total	207	172 (83.1)	NA	NA	44.8 ± 6.0	NA
	CPVI + BOX	102	88 (86.3)	58.9 ± 10.5	NA	45.0 ± 5.3	44.0
	CPVI	105	84 (80.0)	58.6 ± 11.0	NA	44.5 ± 6.7	33.1
Yamaji* et al*. 2020 [10]	Total	78	63 (80.8)	NA	NA	45 ± 5	NA
	CPVI + BOX	24	20 (84.0)	66 ± 11	25.9 ± 4.0	42 ± 5	24
	CPVI	54	43 (80.0)	66 ± 8	22.7 ± 3.2	45 ± 5	24
Pak *et al*. 2020 [11]	Total	114	82 (71.9)	NA	NA	42.0 ± 6.1	NA
	CPVI + BOX	57	42 (73.7)	58.6 ± 11.4	NA	41.4 ± 6.1	24
	CPVI	57	40 (70.2)	61.6 ± 7.8	NA	42.7 ± 6.1	24
Aryana *et al*. 2021 [12]	Total	110	68 (61.8)	NA	NA	44 ± 4	NA
	CPVI + BOX	55	35 (64)	67 ± 8	30 ± 8	44 ± 4	NA
	CPVI	55	33 (60)	70 ± 9	29 ± 6	44 ± 5	NA
Kistler *et al*. 2023 [13]	Total	338	259 (76.6)	NA	NA	45	NA
	CPVI + BOX	170	131 (77.1)	65.7	29.1	46	5
	CPVI	168	128 (76.2)	65.5	28.6	44	5

NA, not available; CPVI, circumferential pulmonary vein isolation; BMI, body mass index; LAD, left atrial diameter; AF, atrial fibrillation.

**Table 2.** **Catheter ablation procedure data**.

Author	Group	Ablation category	Catheter type	Ablation power	AI/LSI	Ablation time (min)	Procedure time (min)	Procedure-related adverse events
Kim *et al*. 2015 [8]	PVI+BOX	RA	Celsius	25–30 W	NA	128.9 ± 37.9	163.1 ± 47.2	NA
	PVI	RA	Celsius	25–30 W	NA	121.7 ± 58.7	154.9 ± 57.1	NA
Lee *et al*. 2019 [9]	PVI+BOX	RA	Smart Toch/Cool flex	30–35 W	NA	89.0 ± 39.3	226.7 ± 63.1	6
	PVI	RA	Smart Toch/Cool flex	30–35 W	NA	71.5 ± 30.6	206.8 ± 77.7	7
Yamaji *et al*. 2020 [10]	PVI+BOX	RA	Cool flex/Flex Ability	25–30 W	NA	NA	NA	2
	PVI	RA	Cool flex/Flex Ability	25–30 W	NA	NA	NA	2
Pak *et al*. 2020 [11]	PVI+BOX	RA	Flex Ability	25–30 W	NA	88.9 ± 25.3	186.2 ± 52.7	1
	PVI	RA	Flex Ability	25–30 W	NA	69.8 ± 15.9	179.1 ± 60.2	3
Aryana *et al*. 2021 [12]	PVI+BOX	CA+RA	Arctic Front Advance+Smart Toch/Flex Ability	NA	NA	51 ± 15	168 ± 34	3
	PVI	CA+RA	Arctic Front Advance+Smart Toch/Flex Ability	NA	NA	29 ± 14	127 ± 40	3
Kistler *et al*. 2023 [13]	PVI+BOX	RA	Cold saline water infusion catheter	≥40 W	Front wall: 500–550 Posterior wall: 350–400	34 ± 21	142 ± 69	6
	PVI	RA	Cold saline water infusion catheter	≥40 W	Front wall: 500–550 Posterior wall: 350–400	28 ± 12	121 ± 57	4

RA, radiofrequency ablation; CA, cryoablation; AI, the ablation index; NA, not available; LSI, lesion size index; PVI, pulmonary vein isolation.

### 3.4 The Outcome Processed by R Version 4.1.2

#### 3.4.1 Recurrence Rate of Atrial Arrhythmia

**Fig. 3.**
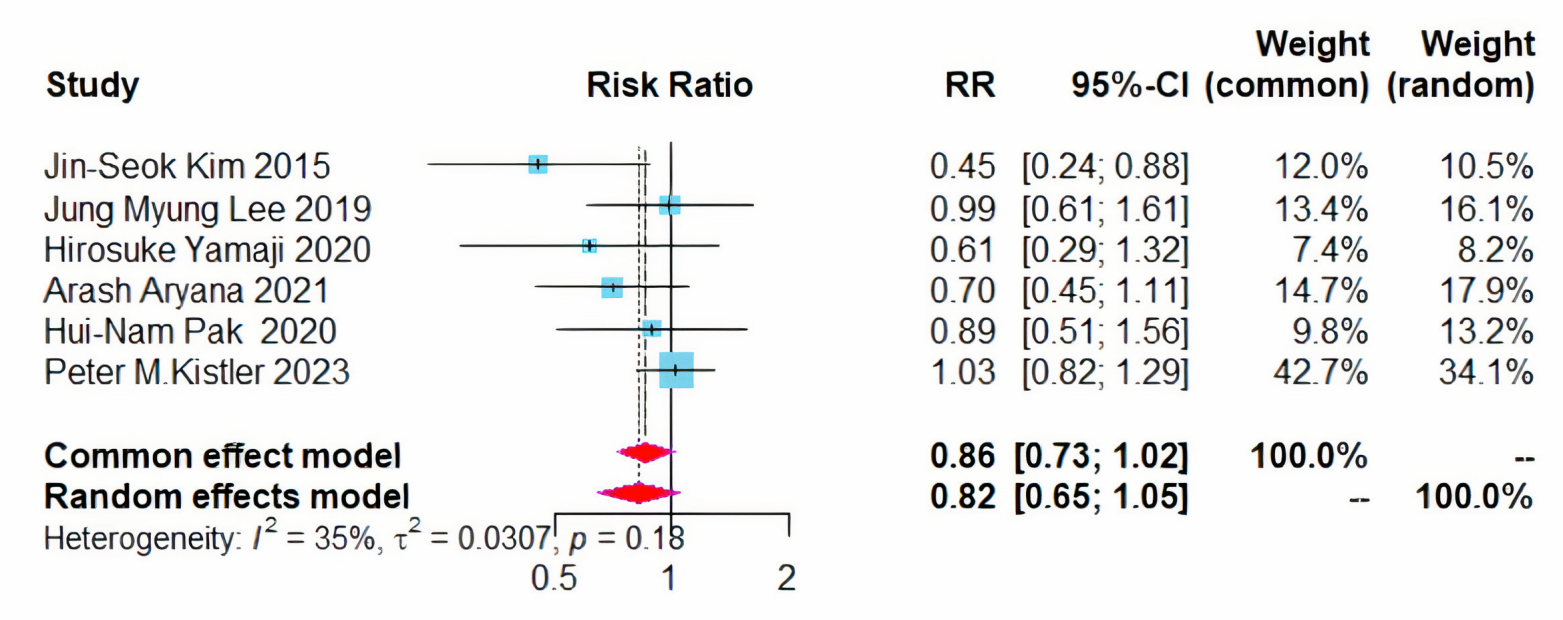
**Forest plot of PVI + BOX versus PVI for all AA recurrence rates**. RR, risk ratio; CI, confidence interval; PVI, pulmonary vein isolation; AA, atrial arrhythmia.

All six studies evaluated the recurrence of atrial arrhythmias post-treatment. The combined approach of CPVI and left atrial BOX ablation did not result in a statistically significant reduction in the overall recurrence rate of atrial arrhythmias, with an RR of 0.86 and a 95% CI ranging from 0.73 to 1.02, while the heterogeneity (I^2^) was 35% (Fig. 3).

**Fig. 4.**
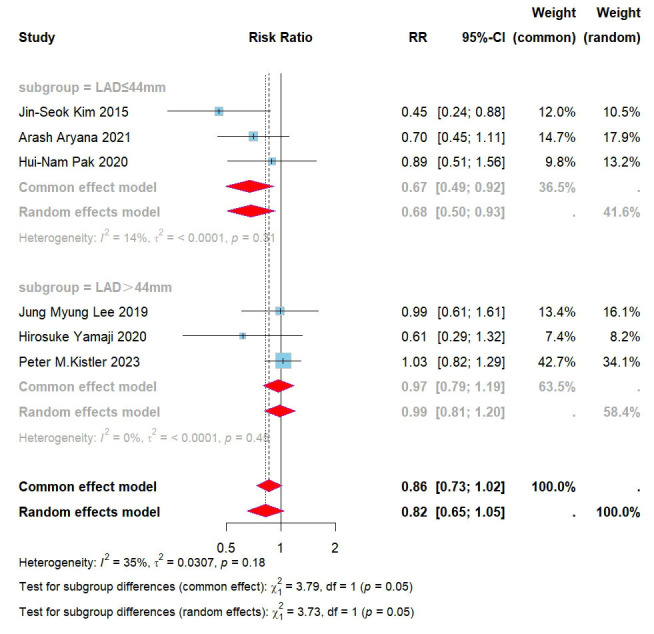
**Forest plot for subgroups of PVI + BOX versus PVI for all AAs**. RR, risk ratio; CI, confidence interval; PVI, pulmonary vein isolation; AAs, atrial arrhythmias; LAD, left atrial diameter.

Given that the 95% CI approached critical values and the heterogeneity (I^2^) was 35%. Sensitivity analyses were conducted using the leave-one-out method, which indicated that the overall results were stable. However, the data from Kistler *et al*. [13] significantly impacted the outcomes. Subsequently, we investigated the sources of heterogeneity further based on the collected data. Based on the surgical experience at our research center, the lowest recurrence rate of arrhythmia during follow-up was observed with CPVI combined with BOX ablation when the left atrial diameter was less than 44 mm. As the left atrial diameter increased, the recurrence rates for both CPVI combined with BOX ablation and CPVI alone increased. Further data analysis revealed that within the study conducted by Kistler *et al*. [13], the average left atrial diameter (LAD) was the largest among patients who underwent CPVI combined with BOX ablation. It is hypothesized that the left atrial diameter is a critical factor influencing the recurrence rate of CPVI combined with BOX ablation, with a proposed threshold value of 44 mm. Based on these findings, subgroup analyses were conducted according to the left atrial diameter. Since individual LAD data were not disclosed in the studies, these analyses used the average LAD of the populations included in each study. The results indicated that for patients with a left atrial diameter ≤44 mm, the combination of CPVI and left atrial BOX ablation markedly reduced the recurrence rate of all atrial arrhythmias in those with persistent atrial fibrillation (RR = 0.67, 95% CI = 0.49–0.92, I^2^ = 15%) (Fig. 4).

#### 3.4.2 Recurrence Rate of Atrial Fibrillation and Atrial Tachycardia/Atrial Flutter

**Fig. 5.**
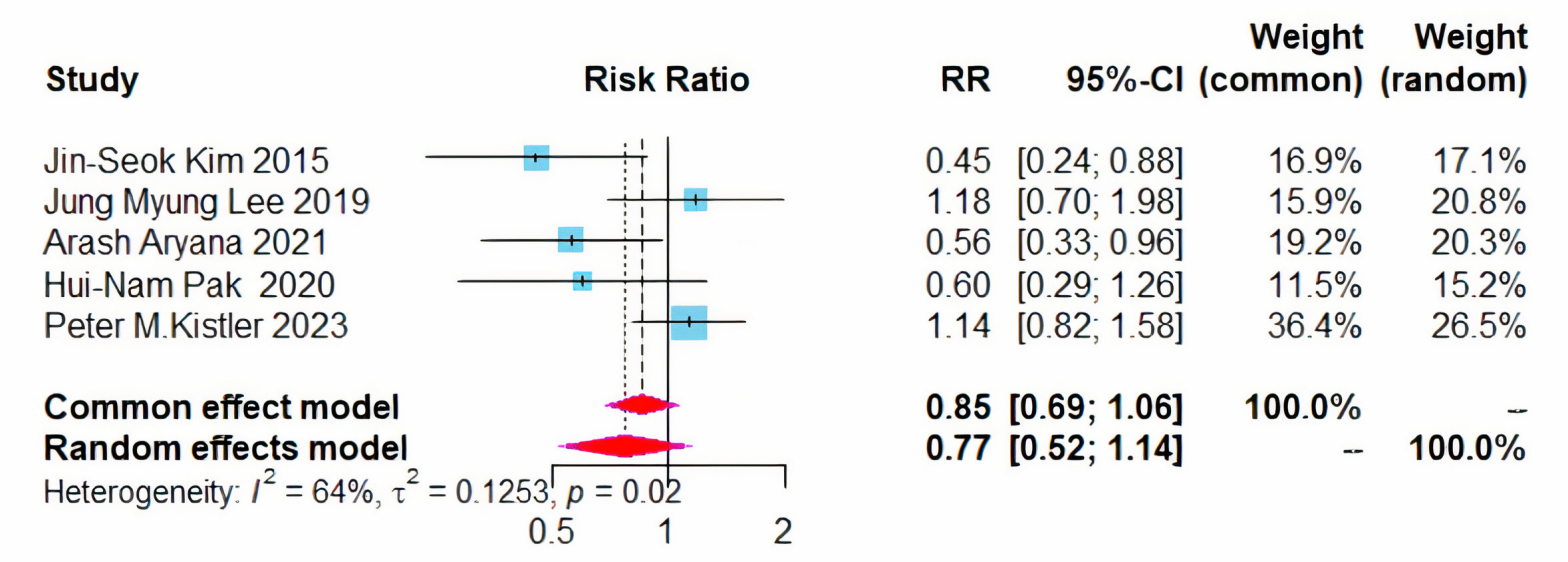
**Forest plot of PVI + BOX versus PVI for AF recurrence rates**. RR, risk ratio; CI, confidence interval; PVI, pulmonary vein isolation; AF, atrial fibrillation.

**Fig. 6.**
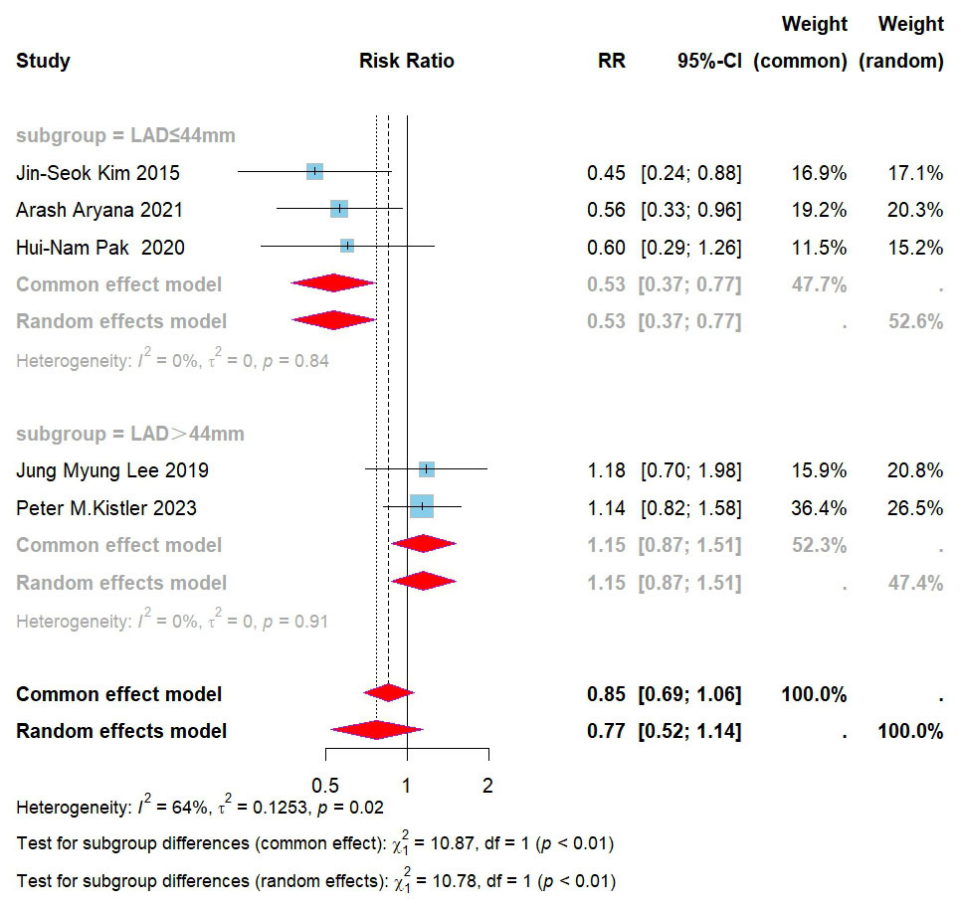
**Forest plot for subgroups of PVI + BOX versus PVI for AF recurrence**. RR, risk ratio; CI, confidence interval; PVI, pulmonary vein isolation; AF, atrial fibrillation; LAD, left atrial diameter.

Five studies assessed the recurrence rates of atrial fibrillation and atrial tachycardia/atrial flutter [8, 9, 11, 12, 13]. No significant difference was observed in the recurrence rates of AF between the two treatment modalities (RR = 0.77, 95% CI = 0.52–1.14, I^2^ = 64%) (Fig. 5). Given the I^2^ exceeded 50%, a sensitivity analysis was conducted, yet no sources of heterogeneity were identified. Further subgroup analysis, based on left atrial diameter, revealed that in patients with a diameter ≤44 mm, CPVI plus left atrial BOX ablation substantially lowered the recurrence rate of moderate atrial fibrillation (RR = 0.53, 95% CI = 0.37–0.77, I^2^ = 0%). However, for diameters >44 mm, no difference in recurrence rate was found between the two approaches in persistent AF cases (RR = 1.15, 95% CI = 0.87–1.51, I^2^ = 0%) (Fig. 6). After conducting subgroup analyses, the consistency across all groups was observed to be 0%.

**Fig. 7.**
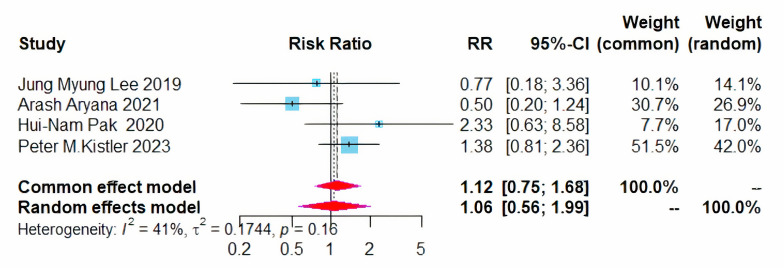
**Forest plot of PVI + BOX versus PVI for AT/AFL recurrence rates**. RR, risk ratio; CI, confidence interval; PVI, pulmonary vein isolation; AT, atrial tachycardia; AFL, atrial flutter.

Additionally, no significant difference was noted in the recurrence of atrial tachycardia/atrial flutter between patients treated with CPVI plus BOX ablation and those receiving CPVI alone (RR = 1.12, 95% CI = 0.75–1.68, I^2^ = 41%) (Fig. 7).

#### 3.4.3 Incidence of Procedure-Related Adverse Events

**Fig. 8.**
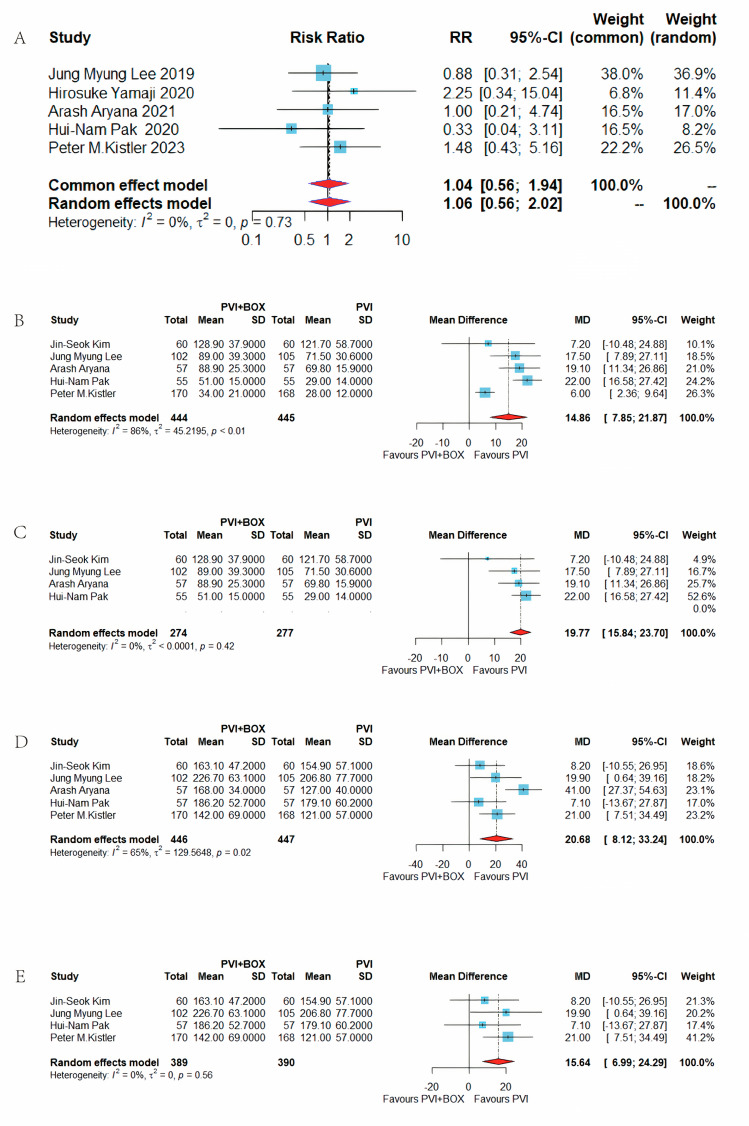
**Comparison of PVI+BOX and PVI: adverse events, ablation time, and procedure time analysis**. (A) Forest plot of PVI+BOX vs. PVI for incidence of adverse events. (B) Forest plot of PVI+BOX vs. PVI for ablation time. (C) Forest plot of PVI+BOX vs. PVI for ablation time after sensitivity analysis. (D) Forest plot of PVI+BOX vs. PVI for procedure time. (E) Forest plot of PVI+BOX vs. PVI for procedure time after sensitivity analysis. RR, risk ratio; CI, confidence interval; PVI, pulmonary vein isolation; MD, mean difference; SD, standard deviation.

Five studies assessed the incidence of procedure-related adverse events. The addition of left atrial BOX ablation to CPVI did not significantly alter the risk of adverse events compared to CPVI alone (RR = 1.04, 95% CI = 0.56–1.94, I^2^ = 0%) (Fig. 8A) [9, 10, 11, 12, 13].

#### 3.4.4 Ablation Duration

The ablation duration was reported in five studies [8, 9, 11, 12, 13]. The integration of CPVI and left atrial BOX ablation extended the ablation time by an average of 14.86 minutes (mean difference (MD) = 14.86, 95% CI = 7.85–21.87, I^2^ = 86%) (Fig. 8B). High heterogeneity (I^2^ > 50%) prompted a sensitivity analysis, which identified the study by Kistler as the source due to its high-power, short-duration strategy, which required less time for additional ablation on the posterior wall. After excluding this study, the adjusted average increase in ablation duration was 19.77 minutes (MD = 19.77, 95% CI = 15.84–23.70, I^2^ = 0%) (Fig. 8C).

### 3.5 Operation Duration

Five studies [8, 9, 11, 12, 13] reported the operation duration, indicating that the combination of CPVI and left atrial BOX ablation lengthened the total operation time by an average of 20.68 minutes (MD = 20.68, 95% CI = 8.12–33.24, I^2^ = 65%) (Fig. 8D). The study by Aryana, which combined cryoablation with RF ablation, was identified as a source of significant heterogeneity (I^2^ > 50%) [12].

After excluding this study, the revised average increase in operation time was 21.00 minutes (MD = 15.64, 95% CI = 6.99–24.29, I^2^ = 0%) (Fig. 8E).

## 4. Discussion

This meta-analysis evaluated six RCTs to compare the efficacy of CPVI combined with left atrial BOX ablation versus CPVI alone in treating persistent atrial fibrillation. The findings indicate that the combined approach did not markedly decrease the overall recurrence rate of atrial arrhythmias post-ablation. However, in subsets of patients with smaller left atrial diameters, this combination significantly reduced the recurrence of atrial arrhythmias and fibrillation. While the combined procedure extended both ablation and overall operation times, it did not elevate the risk of procedure-related complications, thereby affirming the safety and efficacy of left atrial BOX ablation.

**Fig. 9.**
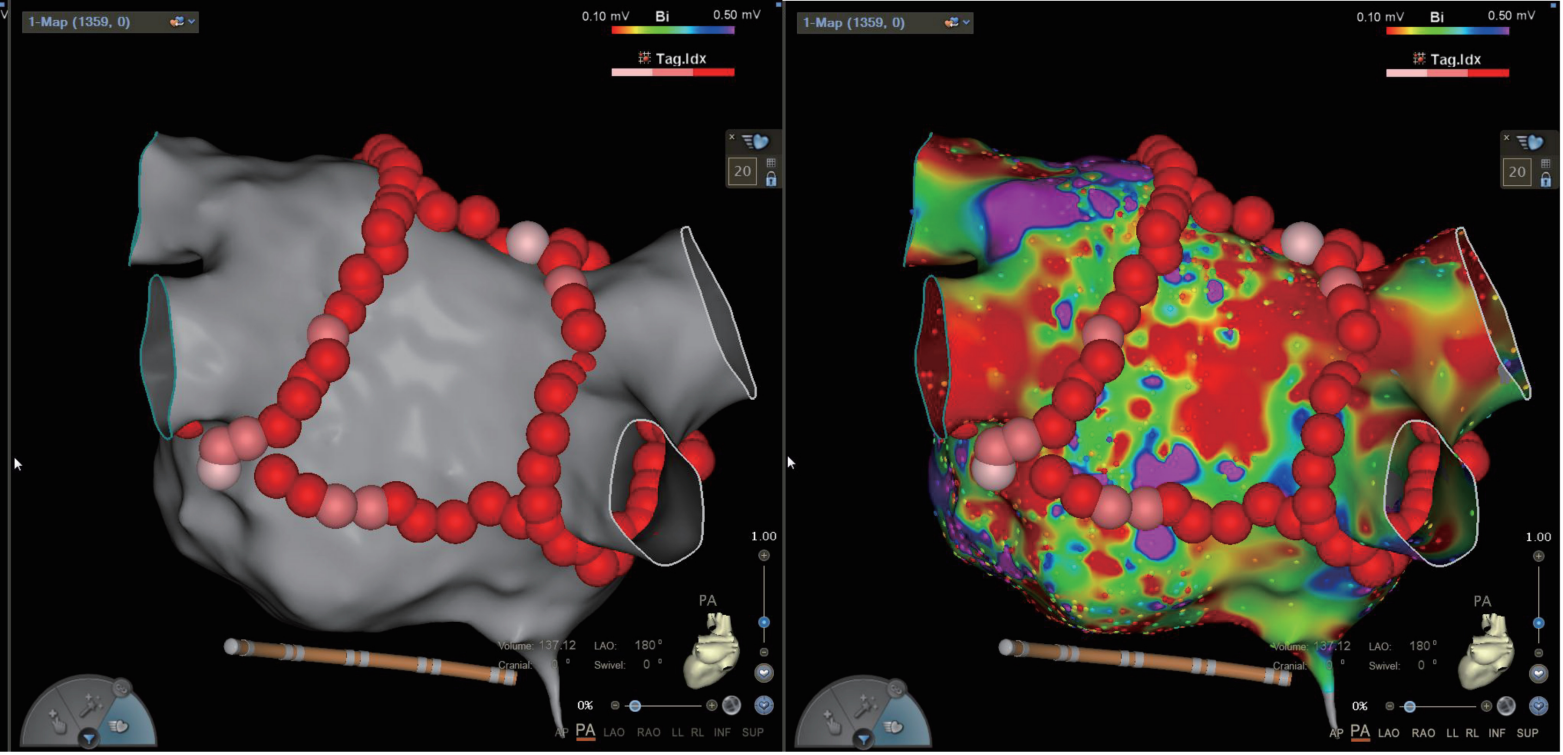
**Image of BOX ablation and EP electroanatomic mapping**. EP, electrophysiology; AP, anterior-posterior; PA, posterior-anterior; LAO, left anterior oblique; RAO, right anterior oblique; LL, left lateral; RL, right lateral; INF, inferior; SUP, superior.

CPVI is established as the foundational ablation technique for atrial fibrillation [5], owing to it being particularly effective in maintaining sinus rhythm in paroxysmal AF patients [14], albeit with a notable recurrence in persistent AF cases, where the recurrence rate post-CPVI has been documented at 43% [15]. Persistent AF patients often exhibit extensive atrial fibrillation and larger left atrial diameters, suggesting additional extrapulmonary triggers and mechanisms. Given the shared embryonic origin of the left atrial posterior wall and the pulmonary veins, ablating the posterior wall is a supported strategy [16]. Catheter ablation in persistent AF frequently reveals low-voltage areas in the posterior left atrial wall, where vagal ganglia plexuses contribute to the electrical and anatomical remodeling, making it a critical site for AF triggers and maintenance [17]. Left atrial BOX ablation, following CPVI, isolates the posterior wall by connecting the superior line, thereby enhancing safety due to its proximity to the esophagus and the presence of a thicker atrial muscle and fat pad [18, 19]. None of the six RCT studies included in this article had an atrial esophageal fistula. We provided images of BOX ablation and electrophysiology (EP) electroanatomic mapping (Fig. 9). The EP electroanatomic mapping was conducted before the ablation.

This study found that additional left atrial BOX ablation in patients with smaller left atrial diameters could effectively lower the recurrence of atrial arrhythmias and AF. The correlation between the increase in left atrial diameter and the duration of AF suggests alternative triggers and mechanisms in post-structural remodeling, influencing the overall recurrence rate. Furthermore, based on our analysis, we can hypothesize that CPVI combined with BOX ablation may be effective in patients with paroxysmal atrial fibrillation. Thus, early detection and intervention could potentially reduce postoperative AF recurrence. Hence, patients with AF are advised to undergo early radiofrequency ablation to prevent left atrial enlargement and ensure a better prognosis and lower recurrence.

Regarding ablation duration, left atrial BOX ablation lengthened the ablation and total procedure times compared to PVI alone. However, the study by Kistler *et al*. [13] demonstrates that additional BOX ablation would not be prolonged with a high power short time course ablation strategy. Moreover, the high-power short-range ablation strategy can reduce the recurrence rate of atrial fibrillation after left atrial BOX ablation [20].

## 5. Limitations

The review included six studies encompassing 967 patients, thereby representing a relatively small sample size. Although the results indicate that CPVI combined with BOX ablation significantly reduces the recurrence rate of arrhythmias in patients with left atrial diameter ≤44 mm, the small sample size may affect the robustness of these findings. Consequently, further research involving a larger cohort is essential. Additionally, it is necessary to conduct studies to confirm the effectiveness of CPVI combined with BOX ablation in patients with larger LAD.

The six studies in this analysis followed CPVI with additional linear ablation, encompassing the mitral isthmus line, tricuspid isthmus line, and anterior wall lines. However, the variability in ablation practices across different centers and among operators precluded a detailed analysis of the impact of these additional ablations on recurrence rates.

Adopting advanced cold saline infusion pressure-sensing ablation catheters, which are now prevalent, was inconsistent due to the varying trial timelines. Consequently, the influence of the catheter selection on the recurrence of atrial fibrillation could not be assessed.

Of the six studies, only the Kistler *et al*. [13] study employed high-power and AI-guided ablation, while the remaining five utilized lower ablation powers and did not report AI parameters at different ablation sites. Additionally, avoiding a bidirectional block on the posterior wall line due to concerns over the risk of atrial–esophageal fistula may have influenced outcomes. Future studies employing high-power, short-duration, AI-guided ablation strategies that achieve a bidirectional block on the modified posterior wall line may demonstrate a lower recurrence rate post-CPVI combined with left atrial BOX ablation.

The primary outcome of the included studies was the recurrence rate of all atrial arrhythmias one year after operation; however, the recurrence rates over longer follow-up periods remain unspecified. Therefore, future research should extend the follow-up duration beyond one year to better assess the long-term outcomes.

## 6. Conclusions

In patients with persistent AF, CPVI combined with left atrial BOX ablation did not substantially reduce the recurrence rate compared to CPVI alone. Nevertheless, for patients with a smaller left atrial diameter, this combined approach significantly enhanced the success of catheter ablation for persistent AF, reducing the recurrence of atrial arrhythmias and fibrillation without increasing the risk of surgical complications. Left atrial BOX ablation proved both effective and safe. Further RCTs with standardized AF ablation protocols are essential to corroborate these results.

## Availability of Data and Materials

The datasets used and analyzed during the current study are available from the corresponding author on reasonable request.

## Abbreviations

AF, atrial fibrillation; AA, atrial arrhythmia; AT, atrial tachycardia; AFL, atrial flutter; CPVI, circumferential pulmonary vein isolation; RCT, randomized controlled trial; CI, confidence interval; RR, relative ratio; WMD, weighted-mean difference; AI, ablation index; LSI, lesion size index.

## Supplementary Material

Supplementary material associated with this article can be found, in the online version, at https://doi.org/10.31083/j.rcm2509316.
